# Factors Affecting Patient Adherence to Multivitamin Intake After Bariatric Surgery: Thematic Analysis of Qualitative Data From a Multicenter Study

**DOI:** 10.7759/cureus.42928

**Published:** 2023-08-03

**Authors:** H.J.M. Smelt, S. Pouwels, L. Heusschen, E.J. Hazebroek, P.W.J. van Rutte, W. Theel, J.F. Smulders

**Affiliations:** 1 Department of Surgery, Catharina Hospital, Eindhoven, NLD; 2 Department of Surgery, Helios Klinikum, Krefeld, DEU; 3 Department of Surgery, Rijnstate, Arnhem, NLD; 4 Department of Human Nutrition and Health, Wageningen University, Wageningen, NLD; 5 Department of Surgery, Onze Lieve Vrouwe Gasthuis (OLVG), Amsterdam, NLD; 6 Surgery, Franciscus Gasthuis, Rotterdam, NLD

**Keywords:** thematic analysis, patient adherence, patient compliance, multivitamin supplement, vitamin supplementation, metabolic surgery, bariatric surgery

## Abstract

Introduction

Adherence to daily intake of multivitamin supplementation (MVS) is a major challenge after bariatric surgery (BS). The aim of this study was to identify insights into patients’ beliefs and experiences on adherence to MVS intake.

Methods

A thematic analysis of qualitative data from four high-volume bariatric centers in the Netherlands was conducted. A series of texts from the open-ended question of 1,246 patients were thematically analyzed for common or overarching themes, ideas, and patterns.

Results

Five key themes emerged regarding participants’ suggestions on adherence to daily MVS intake: “gastrointestinal side effects to MVS intake” (n = 850, 68.2%), “negative features of MVS” (n = 296, 23.8%), “satisfaction with advice on MVS” (n = 272, 21.8%), “dissatisfaction with service provision” (n = 160, 12.8%), and “costs” (n = 93, 7.5%). Most problems were experienced when using specialized weight loss surgery (WLS) MVS. These supplements may cause gastrointestinal side effects, and costs are too high. After bariatric surgery, numerous patients strongly felt that information provision was poor in several aspects, and the aftercare pathway process did not provide sufficient support.

Conclusion

This study found five major themes involved in patient adherence to multivitamin intake after BS: gastrointestinal side effects to MVS intake, negative features of MVS, satisfaction with advice on MVS, dissatisfaction with service provision, and costs of specialized MVS. Challenges lie in stronger education for both patients and healthcare professionals. More personalized care could probably increase patient satisfaction, and MVS companies should look at further optimizing supplements for better tolerability and reducing costs.

## Introduction

Worldwide, the number of individuals undergoing bariatric surgery (BS) for severe obesity and/or associated metabolic diseases is growing rapidly [1,2]. Despite their effectiveness in weight reduction and improved health outcomes, all bariatric surgery procedures alter the anatomy and physiology of the gastrointestinal tract. After surgery, there is an increased risk of developing vitamin deficiencies due to a combination of preexisting deficiencies, decreased postoperative food intake, and reduced absorption in the gastrointestinal tract [3,4]. Therefore, lifelong multivitamin supplementation (MVS) use is recommended, preferably specialized weight loss surgery (WLS) supplements [4-6]. However, a large percentage of patients stop taking MVS or become less consistent with daily MVS intake over time [7,8].

Poor adherence to MVS intake after BS can potentially cause nutritional complications including anemia, bone loss, or even neurological problems. Thus, patient adherence to MVS therapy is a major challenge, and therefore, it is important to understand the reasons behind poor adherence [7-9]. Factors affecting patient adherence to MVS intake after BS were examined in a multicenter questionnaire study by our research group [10]. In this study, 1,200 (26%) patients did not take their MVS consistently or stopped taking MVS [9], which is similar to the systematic review by Zarshenas et al. (20%-32%) [7]. The key reason for cessation or inadequate intake of MVS were gastrointestinal side effects, an unpleasant taste or smell of the MVS, normalized laboratory results, forgetting to take the MVS, and high costs of the supplements [10]. Moreover, many patients were dissatisfied with the information provided by healthcare professionals about MVS and the little attention paid to MVS use during medical consultations. Healthcare professionals often do not ask about patient beliefs or personal preferences. These results emerged from 41 multiple-choice questions in our multicenter study. The present study continues on the obtained results from our patient survey [10] by performing a thematic analysis of the data from one open-ended question. The aim of this study was to get more insights into the patient’s perspective and experiences regarding adherence to MVS intake after bariatric surgery.

## Materials and methods

In this study, a multicenter thematic analysis of qualitative data was done. We conducted an anonymous survey study among patients after bariatric surgery from four high-volume bariatric centers in the Netherlands: Catharina Hospital Eindhoven, Rijnstate Arnhem, Franciscus Gasthuis and Vlietland, and Onze Lieve Vrouwe Gasthuis (OLVG) Amsterdam. The questionnaire consisted of 41 multiple-choice questions and one open-ended question at the end. A previous review by our research group on potential influencing factors that negatively affect adherence to MVS intake was used as input for the questions [8]. In total, 15,424 patients were recruited between October and December 2020. Digital informed consent was obtained from all participants. Due to the large volume of data, the results of the whole survey were divided into two parts. Part 1 consisted of an extensive analysis (n = 4,614) of answers from 41 multiple-choice questions. The results of part 1 have already been published [10]. The present thematic analysis is a description of part 2. This part consisted of an analysis of one open-ended question with unlimited text capability. The open-ended question was as follows: “Do you have any additional comments that may help improve our advice regarding the use of MVS or any other comments at all?” The open-ended question was meant to elicit more comprehensive and detailed responses from the participants. The full answers were used in this analysis. No details have been omitted. Some of the extended responses covered multiple themes and were therefore included in several themes.

We included patients of 18 years and older who underwent bariatric surgery from 2010 to 2020, including sleeve gastrectomy, Roux-en-Y gastric bypass, one anastomosis gastric bypass, single anastomosis duodenal-ileal bypass, duodenal switch, and revisional and/or secondary surgery. Exclusion criteria were incomplete questionnaires or reversal of the bariatric procedure (“undo” surgery).

The data in this thematic analysis was characterized by grouping repeated semantic code patterns into meaningful categories/themes [11,12].

Step 1: Familiarization

The researcher read and studied textual responses, took notes, highlighted important topics, and identified relevant concepts. This step was performed for developing a deep understanding of the content, context, and nuances of the data.

Step 2: Coding

The approach to coding was inductive. Assigning codes and subcodes to specific textual data was done in two steps.

Step 1: Open Coding

Key meanings were coded generating a wide range of possible themes.

Step 2: Selective Coding

Identified themes were organized and refined looking at interrelationships between codes. The coding process was iterative with data being repeatedly analyzed, new codes developed, and existing codes refined. Consultation with the research group was always sought whenever there was any doubt about an answer or theme.

Step 3: Generating themes

After coding, broader thematic categories were formed. Codes and subcodes were connected, after which overarching themes were identified.

Step 4: Reviewing themes

Identified themes and interpretations were critically reviewed to ensure the accuracy, consistency, and reliability of the analysis. The themes were assessed for several aspects, such as relevance and internal consistency, checking that the data assigned to each theme are relevant and representative of that particular theme. Internal consistency within each theme was checked to ensure that the assigned codes and citations are coherent and tell a coherent story. The researcher also looked for connections, overlaps, hierarchies, or contradictions between the different themes to better understand the broader context.

Step 5: Defining and naming themes

The researcher checked whether the definition and name of the theme matched the coded data and accurately reflected the content and meanings. Subsequently, final theme names were formed.

Data management

All data were collected anonymously in Data Management^®^ (Cloud9, Research Manager, Deventer, The Netherlands) and exported into ATLAS.ti^®^ version 22.1 (ATLAS.ti Scientific Software Development GmbH, Berlin, Germany) for qualitative analysis [13].

## Results

In total, 1,387 patients answered the open-ended question, and 141 answers were excluded from this analysis because they were not contributing to the research question or were not related to the study subject. Answers from 1,246 patients were included in this study. The baseline characteristics of all included patients were described in Table 1. Five key themes emerged regarding participants’ suggestions on adherence to daily MVS intake. These themes were “gastrointestinal side effects to MVS intake” (n = 850, 68.2%), “negative features of MVS” (n = 296, 23.8%), “satisfaction with advice on MVS” (n = 272, 21.8%), “dissatisfaction with service provision” (n = 160, 12.8%), and “costs of the WLS MVS” (n = 92, 7.5%).

**Table 1:** Baseline characteristics of the included patients

Baseline characteristics	Subgroups	Total group (N = 1,246)
Age (years)		50.1 ± 10.5
Marital status (number)	Single	179 (14.4%)
	Living with partner	164 (13.2%)
	Married or registered partnership	803 (64.4%)
	Divorced or separated	72 (5.8%)
	Widowed	28 (2.3%)
Education level (number)	Low	703 (56.4%)
	Middle	513 (41.2%)
	High	81 (6.5%)
Body weight (kg)		87.1 ± 18.4
Body mass index (kg/m^2^)		29.8 ± 5.5
Type of surgery (number)	Sleeve gastrectomy	442 (35.5%)
	Roux-en-Y gastric bypass	708 (56.8%)
	One anastomosis gastric bypass	23 (1.9%)
	Revision surgery	60 (4.8%)
	Unknown	13 (1%)
Time since surgery (number)	0-1 years	173 (13.9%)
	1-2 years	273 (21.9%)
	2-3 years	292 (23.4%)
	3-4 years	276 (22.2%)
	4-5 years	145 (11.6%)
	>5 years	87 (7%)

Theme 1: Gastrointestinal side effects of MVS intake (n = 850, 68.2%)

Gastrointestinal side effects related to MVS intake were the predominant theme in this analysis. Nausea was the most commonly reported side effect (n = 236, 18.9%) and led to vomiting in some patients (n = 55, 4.4%). This was followed by stomach or abdominal pain (n = 110, 8.8%). Other complaints that occurred less often are gastroesophageal reflux disease, belching, or burping (n = 44, 3.5%), dumping (n = 8, 0.6%), hunger (n = 3, 0.2%), diarrhea (n = 7, 0.6%), flatulence (n = 3, 0.2%), and constipation (n = 1, 0.1%). Furthermore, there were many patients with general malaise or gastrointestinal symptoms, which were not specified in the answer (n = 87, 7%). Besides that, 296 (23.8%) patients experienced gastrointestinal complaints by basic features of the supplements as described in detail in theme 2. The following are a few quotes from the patients: “The vitamins make me nauseous and caused vomiting, and they get stuck in my throat every day; those symptoms are worse than the disadvantages of not taking the vitamins”; “It causes nasty acid regurgitation and vomiting”; and “I get nauseous when I open the vitamin jar.”

Theme 2: Negative features of MVS (n = 296, 23.8%)

This theme described how the MVS composition can be a problem for the tolerability of the supplements in many patients. Specialized WLS MVS capsules as well as chewable tablets were the most commonly reported formulas. Many patients experienced complaints such as nausea or emesis due to the size (n = 92, 7.4%) or smell (n = 39, 3.1%). The smell of the WLS MVS was described as foul and repulsive, which led to feelings of nausea and triggered the gag reflex (n = 8 for WLS capsule, n = 2 for WLS chewable, and n = 29 for unknown). Patients reported the taste as bad (n = 165, 13.2%), because of the sweet taste (n = 14, 1.1%) or sour taste (n = 4, 0.3%), dumping complaints (n = 5, 0.4%), and a very nasty aftertaste or halitosis that lasted for hours (n = 147, 11.8%). MVS users of regular “over-the-counter” MVS reported no problems with the taste of their supplement. Some patients experienced swallowing difficulties and a sensation of the MVS being stuck in the throat (n = 70 for WLS capsule, n = 21 for WLS chewable tablets, and n = 1 for regular MVS). The following are a few quotes from the patients: “The awful sweet taste is very annoying to me because it causes diarrhea, nausea, and usually dumping”; “The capsules are too big and therefore get stuck in my throat, causing nausea and vomiting, and the capsule feels too heavy on my stomach”; and “The taste and smell are really disgusting and unacceptable because it causes nausea.”

Theme 3: Satisfaction with advice on MVS (n = 272, 21.8%)

This section was divided into three subthemes: administration of MVS, managing potential nutrition interactions, and information provision about laboratory results. Quotes from patients are also described in quotation marks below each paragraph.

Administration of MVS (n = 104, 8.4%)

In the past, only one brand of WLS MVS was prescribed in many obesity centers in the Netherlands. Many patients reported being dissatisfied with this advice and aspired to alternative options for various reasons such as personal circumstances or preferences (e.g., costs and side effects). Six of them hoped that WLS MVS could be administered in injection form with a less frequent schedule in the future. The following are a few quotes from the patients: “It is annoying that only one brand of WLS MVS is prescribed and I cannot choose the brand myself; I don’t like that I can’t try different types of MVS”; “I can decide for myself what kind of MVS I want to use; there is too much pushing for WLS MVS, and I don’t like that, and if you don’t use the prescribed WLS MVS, there is no proper guidance”; and “I notice that there is more need for personal guidance; in addition, the use of supplements should be more personalized.”

Managing Potential Nutrient Interactions (n = 119, 9.6%)

Many patients described problems with the timing of taking the MVS. The most commonly cited reason was that the MVS had to be taken separately from calcium supplements or calcium-rich foods. Most patients did not really know what to do in practice and frequently forgot to take calcium. Additionally, they received no or only limited advice about this subject. Some patients were not aware that separate intake was necessary or had never been advised to take calcium. The less frequently cited reasons were the insufficient support for the interaction of MVS or calcium supplements with other medications. Patients did not know the best time to take the supplements due to this interaction. The following are a few quotes from the patients: “The fact that you have to pay attention to what time you take the MVS and calcium is a major disadvantage”; “I take 15 pills a day and don’t know how it interacts with MVS”; and “I regularly turn into problems with the intake of the MVS because there must be two hours between intake of calcium tablets but also the same time in combination with dairy products.”

Information Provision About Laboratory Results (n = 49, 3.9%)

The dose of the MVS could be adjusted based on laboratory results. In this case, the dose of MVS intake was reduced on the advice of general practitioners, internal medicine specialists, or professionals from the obesity center. The most frequently reported obstacle was that healthcare professionals only mentioned the reduction of the MVS dose without providing an explanation about hypervitaminosis. Furthermore, some patients described that healthcare professionals did not offer any suggestions for customized MVS options. As a result, patients took the initiative to reduce the MVS dose themselves. The following are a few quotes from the patients: “I had to figure out by myself why my vitamin B6 levels were alarmingly high and switched to another MVS myself” and “It’s unfortunate that doctors don’t really think about MVS and changing blood levels and have little insight into this.”

Theme 4: Dissatisfaction with service provision (n = 160, 12.8%)

Dissatisfaction is divided into three subthemes: dissatisfaction about the postoperative care pathway, dissatisfaction about not feeling heard, and dissatisfaction about WLS MVS companies. Quotes from patients are also described in quotation marks below each paragraph.

Dissatisfaction About Postoperative Care Pathway (n = 65, 5.2%)

The postoperative outpatient clinical care was performed by a multidisciplinary team for five years. The follow-up of all included hospitals in this study was similar. Patients were dissatisfied with the duration and frequency of the aftercare program. They particularly experienced good support in the first year and only limited guidance from the second to fifth year (n = 51, 4.1%). The negative attitude toward aftercare during the corona pandemic was striking (n = 14, 1.1%). Most face-to-face consultations were converted to online consultations, which caused dissatisfaction. The short consultation time of 10-15 minutes yearly was also described as inadequate. Some patients did not think the high healthcare costs for a 10-minute consultation including a blood test were worth it. Several patients also indicated that postoperative group meetings were not suitable for them. They did not feel heard or did not dare to discuss their problem, fearing it would cause trouble in their self-confidence when another patient lost much more weight. However, the same postoperative care pathway was offered to everyone. Patients experienced this as unpleasant and described it as “factory work at the assembly line.” The following are a few quotes from the patients: “I also no longer participate in the annual meeting because the emphasis is on the group discussion, which has no added value for me; the accompanying consultation is very short and, in my opinion, serves more to collect data for the attending physicians than for me as a patient”; “I find the hospital’s attention after the surgery too limited; it’s almost like assembly line work in a factory with no personal attention”; and “In the first two years, there was a lot of aftercare; since then, I come for a checkup of 10 minutes a year and pay my full deductible of €385, and I don’t think this is worth it.”

Dissatisfaction About Not Feeling Heard (n = 68, 5.5%)

Some patients felt unheard during the consultations at the obesity center. The most frequently reported reason was that gastrointestinal complaints and the request to switch to another type of MVS were not taken seriously (n = 26, 2.1%). The follow-up consultations did not meet their needs or preferences. Other less frequently reported reasons were weight regain (n = 23, 1.9%), excess skin surplus (n = 14, 1.1%), unrecognized vitamin B12 deficiency (n = 3, 0.2%), and disturbed eating behavior or alcohol addiction (n = 2, 0.2%). Patients missed personal attention, personalized MVS advice, and sufficient time to discuss gastrointestinal complaints or personal questions. Psychological counseling was inadequate, and patients often did not feel comfortable bringing up mental health issues themselves. Patients missed a standard checkup with a plastic surgeon or were disappointed that skin correction procedures were not reimbursed despite successful weight loss. The following are a few quotes from the patients: “I found the approach of the doctors to be negative; they only look at tables and whether you are a success story, and if you are not, then they are downright negative and unfriendly, and this does not encourage patients to keep coming to appointments; I experience many gastrointestinal complaints, but they are not taken seriously, and now I go to the general practitioner, at least he listens”; “For me, as a young adult, it is difficult to deal with so much weight loss, because of the excess skin on my belly; this makes me sad, and little attention is paid to excess skin in the preoperative process”; and “If you have symptoms, not enough attention is paid to it, and laymen within social media forums give incorrect advice to each other; therefore, they should ask more questions during consultations, and I hope they will also be open to other MVS brands and personal ideas about them.”

Dissatisfaction About the WLS MVS Companies (n = 27, 2.2%)

It was notable that patients believed that the obesity center had shares in WLS MVS or that there was commercial interest in using WLS MVS. Patients frequently reported difficulties in canceling a subscription with the WLS MVS company due to rigidity and pushiness. After surgery, patients were sometimes contacted earlier by the WLS MVS producer than by the professionals of the obesity center, which was perceived as disruptive. The following are a few quotes from the patients: “Stubbornness of the supplement manufacturer’s customer service; as a medical institution, do you want to commit to this?” “The idea of the WLS MVS is advice from a commercial point of view; I suspect the doctor has special ties to WLS MVS or shares by the company”; “I think they have an aggressive way of selling, and canceling the subscription took a lot of effort on my part, and I found that unpleasant”; and “The subscription gives an uncomfortable feeling; if you point this out, it is hardly accepted, and you are not understood, and the customer service almost forces you not to leave, and there are frequent calls.”

Theme 5: Costs of the WLS MVS (n = 93, 7.5%)

Many patients considered the costs of the WLS MVS to be a problem as they could not afford these supplements. Consequently, patients were tempted to use supplements from other brands or regular over-the-counter MVS that were more affordable for them. The majority of patients believed that it is crucial that specialized WLS MVS be covered by healthcare insurance. The following are a few quotes from the patients: “I like to do what’s best for me, but I don’t take specialized WLS MVS, because the costs are way too high for me”; “I’ve felt great for years without taking that expensive WLS MVS”; and “It’s important that the MVS doesn’t stay so expensive, because then many patients, like me, start looking for alternatives.”

## Discussion

This is the first thematic analysis on adherence to MVS intake after BS. This qualitative study offers rich and compelling insights into patient beliefs, experiences, and perspectives on MVS intake after BS. Five themes emerged regarding adherence to MVS intake: gastrointestinal side effects to MVS intake, negative features of MVS, satisfaction with advice on MVS, dissatisfaction with service provision, and high costs of the specialized WLS MVS. There is no doubt that adherence to MVS intake after bariatric surgery is a multifactorial problem, and our study indicates several points for improvement. The specialized WLS MVS needs to be further optimized by manufacturers to increase tolerability. A proper formula of supplements is necessary to ensure adequate absorption. This requires consideration of all drug substances and pharmaceutical ingredients, which is a major challenge after BS due to anatomical alterations [14]. Ideally, costs should be reduced to make WLS MVS accessible for more patients, and reimbursement should be considered.

In the field of healthcare after BS, numerous patients strongly felt that information provision was poor in several aspects. Particularly, the general information provided about using WLS MVS was insufficient. Patients missed explanations about different WLS MVS options, side effects or gastrointestinal symptoms, the proper way of taking the supplements, and the associated benefits and costs. Healthcare professionals are often not aware of patients’ experience with MVS intake. Moreover, gastrointestinal complaints were not always checked or recognized. It appeared that healthcare professionals lacked sufficient knowledge about the subject or did not thoroughly listen to the patients to provide adequate advice [15,16]. Additionally, patients experienced concerns about whether healthcare professionals had the experience and skills to interpret micronutrient laboratory results and make appropriate adjustments to the MVS. This study reported that the dose of MVS was reduced on the advice of general practitioners, internal medicine specialists, or professionals at obesity centers. However, most healthcare professionals receive very little education in nutrition and the treatment of micronutrient deficiencies after bariatric surgery. Furthermore, many patients reported that the aftercare pathway process did not provide sufficient support. The dissatisfaction appeared to stem from the fact that standard follow-up was offered and tailored care remained insufficient.

The findings from this thematic analysis suggest that patients require more support from healthcare professionals who possess the necessary skills and experience to provide personal-centered care (PCC). PCC would benefit from greater care customization, but first, there needs to be a better understanding of the experience of illness and of how to address patients’ needs within complex and fragmented healthcare delivery systems [17]. Possibly, the greatest need for education is among healthcare professionals in the management of patients who have undergone bariatric surgery.

Postoperative consultations with healthcare professionals are an integral part of patients’ journey regarding multivitamin supplementation. These consultations usually focus on directive communication involving primarily closed questions to obtain necessary information from patients. However, non-directive communication with open-ended questions can encourage patients to express their preferences and barriers to MVS use [18]. Healthcare professionals face challenges to obtain certain necessary information from patients in a limited amount of time. Implementing shared decision-making into routine clinical practice can be helpful to provide PCC with a way to improve patient satisfaction and treatment adherence [18].

Strengths

A major strength of this study was the large sample size, and all patients who had undergone BS between 2010 and 2020 from various high-volume centers in the Netherlands were recruited to reduce selection bias. Participation was anonymous and carried no risk or personal benefit, which minimized the likelihood of socially desirable answers being given. BS is a treatment that requires patient adherence and motivation. In order to achieve this, a shared decision-making process is necessary to enable the lifestyle maintenance that is required for the full benefits of the surgery. Thus, the involvement of patients in bariatric studies is crucial [19,20]. Patient involvement in this thematic analysis primarily focuses on ensuring patients are given a voice to share their experiences and knowledge with healthcare professionals. The insights from this thematic analysis are very valuable and can be used to improve healthcare after BS.

Limitations

This study had all the limitations inherent in qualitative research due to the well-known biases associated with subjective reporting. The study was conducted anonymously, preventing the researcher from contacting the participating patients for any clarification of their answers. There were instances where information about the context in which the described events occurred was missing, potentially leading to misinterpretation by the researcher. Patients who had complaints related to the research topic were more likely to complete the questionnaire than those without such complaints, introducing information bias. Other limitations of qualitative research included difficulties in establishing causality and limitations in generalizability. Furthermore, only one question was analyzed in this study, which was part of a broader research investigation. Nevertheless, the results provided valuable insights into patients’ phenomenological experiences on the subject. Future studies could benefit from conducting interviews and focus groups with participants to obtain more extensive data.

## Conclusions

This study identified five major themes related to patient adherence to multivitamin intake after BS: gastrointestinal side effects to MVS intake, negative features with MVS, satisfaction with advice on MVS, dissatisfaction with service provision, and costs of specialized WLS MVS. The specialized WLS MVS should be further optimized by manufacturers to enhance tolerability and reduce costs. However, the main challenges lie in improving the care pathway within obesity centers by healthcare professionals. Medical consultations should be organized in a way that focuses more on what the patient wants to discuss, rather than simply checking off a medical list in the electronic patient file. Stronger education for healthcare professionals is necessary, and communication skills should be improved. Giving attention to the patient’s story is crucial to providing more personalized aftercare. More personalized aftercare has the potential to increase patients, which is probably the key to improving adherence to MVS intake. There is no doubt that adherence to MVS intake after BS is a multifactorial problem, and a multifactorial approach is essential.
